# Editorial: Artificial intelligence: new hope for critically ill cardiovascular patients

**DOI:** 10.3389/fmed.2024.1453169

**Published:** 2024-07-23

**Authors:** Li-li Wu, Bo-ran Yang, Xin-yi Meng, Guan-wei Fan, Bing Yang

**Affiliations:** ^1^Department of Health Service, Logistics University of People's Armed Police Force, Tianjin, China; ^2^Tianjin Zhongshan Elementary School, Tianjin, China; ^3^Tianjin Wutong High School, Tianjin, China; ^4^Department of Cell Biology, College of Basic Medical Sciences, Tianjin Medical University, Tianjin, China; ^5^National Clinical Research Center for Chinese Medicine Acupuncture and Moxibustion, First Teaching Hospital of Tianjin University of Traditional Chinese Medicine, Tianjin, China; ^6^State Key Laboratory of Modern Chinese Medicine, Key Laboratory of Pharmacology of Traditional Chinese Medical Formulae, Ministry of Education, Tianjin University of Traditional Chinese Medicine, Tianjin, China; ^7^Department of Public Health, International School, Krirk University, Bangkok, Thailand

**Keywords:** editorial, artificial intelligence, cardiovascular disorder, critically ill patients, cardiovascular medicine

Cardiovascular disease (CVD) remains a major challenge for researchers, clinicians, and patients and is a leading cause of morbidity and mortality around the world (1). CVD is a complex and integrated collection of diseases of the heart, vascular system, and blood. Symptoms of CVD may not be limited to the heart itself. They could include all symptoms of narrowed or clogged blood arteries that may affect the muscles, valves, etc. (2). It is a highly personalized condition that is constantly and rapidly changing. This becomes more complex when dealing with critically ill patients.

For years, scientists have been trying to use all the information available to build more accurate, more efficient, and simpler scoring models. Then they gather evidence to prove whether they are better. Thanks to the development of medical tools, we can collect more data than ever before. But that makes it difficult for the physician to process the vast amount of data to make the right decision. This is vitally important for people with cardiovascular disease. In the high-stakes intensive care unit (ICU) environment, the ability to accurately predict patient outcomes is also paramount.

Almost 80 years after the first artificial neural network, artificial intelligence (AI) has been able to prove itself with its powerful capabilities, especially in the field of medicine (3) (Figure 1). Optimized computer infrastructures are attempting to emulate the capabilities of the human brain and offer support to the doctor, especially in the processing of huge amounts of data. By effectively interpreting vast amounts of clinical data, AI is now accelerating the pace of medical development. Cardiovascular medicine is rapidly embracing AI (4). In this historic change, we have been able to find more and more AI-based modalities, such as electrocardiograms and cardiac angiography, in the clinical management and diagnosis of CVD.

**Figure 1:**
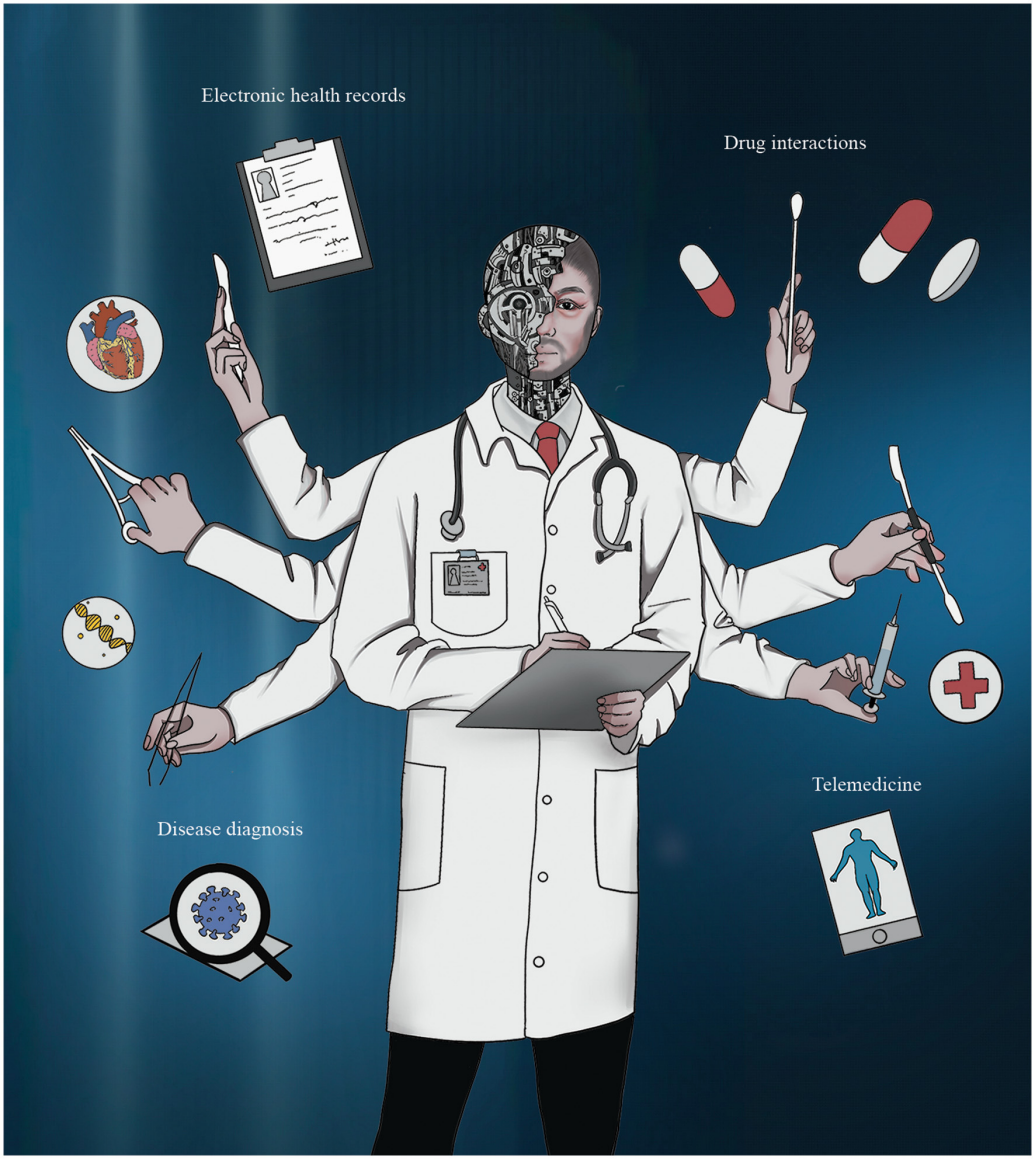
Artificial intelligence changing the future of healthcare. Harnessing the computational power of artificial intelligence to improve medical practice, research, and decision-making. Ultimately, this will lead to more accurate diagnoses, personalized treatments, and improved patient outcomes.

This editorial introduces the collection of articles published in Frontiers in Medicine. We hope that this Research Topic will be able to promote high-quality research on AI in the field of cardiovascular medicine. The following are the researches in the Frontiers in Medicine: Intensive Care Medicine and Anesthesiology collection.

*Analysis of Hematological Indicators via Explainable Artificial Intelligence in the Diagnosis of Acute Heart Failure: A Retrospective Study*, by Yilmaz et al.. Using AI, this study focuses on finding new hematological indicators for acute heart failure. The findings suggest that older age, low platelet count, high erythrocyte distribution width standard deviation, and platelet distribution width are the most important hematology parameters for diagnosing AHF.

*A quasi-experimental study of fresh oxygen flow on patients' oxygen reserve during mask-assisted ventilation under general anesthesia induction* by Shi et al.. Anesthesia is critical for critically ill cardiovascular patients. The different levels of pre-oxygenation in routine clinical anesthesia are compared in this study.

*A Novel Web-based Calculator to Predict 30-Day All-Cause In-hospital Mortality for 7202 Elderly Patients with Heart Failure in ICUs: Multicenter retrospective cohort study in the US* by Wang et al.. They developed a web-based program to predict 0-day mortality in older HF patients in the ICU. Especially for elderly patients admitted to intensive care units. This study also predicts risk factors for in-hospital mortality.

*Randomized Trial on the Effects of Mindfulness-Based Respiratory Decompression Therapy in Claustrophobia Patients Undergoing MRI Inspection* by Zhou et al.. This study concerns the psychological problems that arise in magnetic resonance imaging machines. They found that mindfulness breathing techniques can help patients with claustrophobia complete an MRI scan.

*Development and Validation of Risk Prediction and Neural Network Models for Dilated Cardiomyopathy Based on WGCNA* by Yu et al.. To discover potential biomarkers and therapeutic options for dilated cardiomyopathy, the author developed a prediction model and a neural network. In combined and validation datasets, their results show good accuracy and sensitivity.

*Automating Venous Thromboembolism Risk Assessment: A Dual-Branch Deep Learning Method Using Electronic Medical Records* by Yang et al.. In the case of VTE, a common CVD, the researcher proposed a two-stage deep learning assessment method, finding an intelligent way to assist the physician in a tedious assessment.

*VNN1 as a Potential Biomarker for Sepsis Diagnosis and its Implications in Immune Infiltration and Tumor Prognosis* by Guan et al.. The study looked for biomarkers of sepsis, a serious condition that can be life-threatening in older people. They found that VNN1 could be used as a potential biomarker to help diagnose sepsis. Identifying immune infiltration in tumor tissue to predict a tumor prognosis is also a useful approach.

## Author contributions

L-lW: Writing – original draft. B-rY: Writing – original draft, Visualization. X-yM: Funding acquisition, Writing – original draft. G-wF: Writing – review & editing. BY: Writing – review & editing.
